# Efficient Nyström-type method for the solution of highly oscillatory Volterra integral equations of the second kind

**DOI:** 10.1371/journal.pone.0295584

**Published:** 2023-12-14

**Authors:** Qinghua Wu, Mengjun Sun

**Affiliations:** Hunan University of Science and Engineering, Yongzhou, Hunan, China; University of Porto Faculty of Engineering: Universidade do Porto Faculdade de Engenharia, PORTUGAL

## Abstract

Highly oscillatory Volterra integral equations are frequently encountered in engineering applications. The Nyström-type method is an important numerical approach for solving such problems. However, there remains scope to further optimize and accelerate the Nyström method. This paper presents a novel Nyström-type method to efficiently approximate solutions to second-kind Volterra integral equations with highly oscillatory kernels. First, the unknown function is interpolated at Chebyshev points. Then the integral equation is solved using the Nyström-type method, which leads to a problem of solving a system of linear equations. A key contribution is the technique to express the fundamental Lagrange polynomial in matrix form. The elements of the matrix, which involves highly oscillatory integrals, are calculated by using the classical Fejér quadrature formula with a dilation technique. The proposed method is more efficient than the one proposed in the recent literature. Numerical examples verify the efficiency and accuracy of the proposed method.

## 1 Introduction

Highly oscillatory Volterra integral equations of the second kind, which can be described as follows, are frequently encountered in several engineering applications, including wave propagation, quantum mechanics, acoustic scattering, signal processing, and image recognition.

$$\begin{array}{c} y\left(x\right)+\int_{a}^{x}K\left(\omega \left(x-t\right)\right)y\left(t\right)dt=f\left(x\right),\;x\in \left[a,b\right],\;0\neq \omega \in R, \end{array}$$ (1)

where *y*(*x*) is the unknown function, *f*(*x*) is a given function defined on [*a*, *b*], and *K*(*x*, *t*) is a known highly oscillatory function defined on *D* = {(*x*, *t*): *a* ≤ *t* ≤ *x* ≤ *b*}, which depends on a real positive fixed parameter *ω*. Examples of such kernels are

$$\begin{array}{c} K\left(\omega \left(x-t\right)\right)=\text{exp}\left(i\omega \left(x-t\right)\right)\;\;\text{and}\;\;K\left(\omega \left(x-t\right)\right)=J_{\nu }\left(\omega \left(x-t\right)\right), \end{array}$$

where *J*_*ν*_ is the Bessel function of the first kind of order *ν* ≥ 0.

When *w* ≫ 1, the kernel function is highly oscillatory. Discretizing the integral equation using the collocation or Galerkin methods will involve the calculation of the integral of a highly oscillatory function. Collocation and Galerkin methods based on traditional quadrature formulae are computationally costly, which makes it extremely difficult to solve the integral Eq (1) numerically. The computation of the integrals of kernel functions and unknown functions, along with the discretization of integral equations, are the main challenges in solving integral equations. To address this problem, many methods have been proposed for the calculation of integrals of highly oscillatory functions. These methods include asymptotic methods, Filon-type method, Levin-type method, numerical steepest descent and generalized quadrature rules [1–8]. All these methods have the feature that the higher the frequency, the higher the calculation accuracy. But for general oscillators, the above methods are sometimes complicated.

In order to meet the demand of the application of highly oscillatory integral equations, many scholars have proposed some numerical methods for solving these kinds of integral equations by combining the methods for calculating the highly oscillatory integrals with the collocation methods and the discontinuous Galerkin et al., and providing the error estimates of the numerical methods. In 2004, Davies and Duncan derived the stability and convergence of the solution of the collocation method for the first kind of Volterra integral equation with highly oscillatory Bessel kernel [9]. In 2009, Brunner et al. investigated the discontinuous Galerkin method for solving a class of highly oscillatory integral equations. In 2011, Wang and Xiang et al. studied the numerical solution of a class of highly oscillatory Volterra integral equations by combining asymptotic methods and Filon-type methods for the calculation of highly oscillatory integrals, and gave an error analysis [10]. In 2013, Xiang and Brunner studied the collocation method to solve the weakly singular highly oscillatory integral equation with Bessel kernel, analyzed some asymptotic properties of the solution of the equation, and proposed an efficient algorithm to solve the integral equation numerically [11]. Meanwhile, Xiang and Wu investigated the numerical solution of a class of Volterra integral equations with highly oscillatory triangular kernel by combining the Filon method and the collocation method, and gave an error analysis [12]. In the same period, Xiang and He investigated the discontinuous Galerkin method for solving this class of integral equations and found that the computational accuracy of the discontinuous Galerkin method is comparable to that of the collocation method, but the collocation method is simpler [13]. In 2014, Wu studied the case of solving the weakly singular highly oscillatory Volterra integral equation by the collocation method on graded meshes. It was found that for weak singularities, the graded meshes is effective at low frequencies, while in the case where the kernel function is highly oscillatory, the employed graded meshes are not very useful [14]. Meanwhile, Brunner studied the properties of highly oscillatory Volterra integral operators from a theoretical point of view [15]. In 2015, Ma and Fang et al. studied the direct Filon method for the same kind of integral equations and gave a better error estimation formula [16]. In 2015, Brunner H., Ma Y., Xu Y. studied the highly oscillatory nature of the solutions of highly oscillatory integral differential equations [17]. In 2018, Xiang S et al. proposed a numerical solution to a class of highly oscillatory delay potential integral equations by combining the collocation method with the traditional fast multipole method [18]. In 2019, Zhang Q., Xiang S. combined the collocation method with the traditional fast multipole method to present the numerical solution of a class of highly oscillatory Volterra integral equations and gave error estimates. And it concluded that the fast multipole method can greatly improve the efficiency of the collocation method as the number of collocation points increases [19]. In 2021, using the Block-FMM method based on Chebyshev polynomial expansion, Wu et al. proposed an efficient method for solving the Volterra integral equation and gave error estimates [20]. In 2022, Longbin Zhao et al. derived a method for solving the highly oscillatory Volterra integral equation and analyzed the asymptotic behavior of the solution to obtain an error estimation formula with respect to both frequency and step size [21, 22].

Recently, by combining the Legendre polynomials and Gauss-Legendre quadrature formulas, Luisa et al. presented a Nyström-type method for the numerical approximation of the solution of Volterra integral Eq (1), and the convergence of the method was derived. This new numerical method does not use the method of computing highly oscillatory integrals [23].

In this paper, we combine Chebyshev polynomials with the Nyström-type method to study the numerical solution of the following Volterra integral equations of second kind with highly oscillatory kernel

$$\begin{array}{c} y\left(x\right)+\int_{-1}^{x}K\left(\omega \left(x-t\right)\right)y\left(t\right)dt=f\left(x\right),\;x\in \left[-1,1\right],\;\omega \gg 1. \end{array}$$ (2)

where *y*(*x*) is the unknown function, *f*(*x*) and *K*(*ω*(*x* − *t*)) are given functions. For the general interval [*a*, *b*], it can be mapped to the interval [−1, 1] by the transformation $t=a+\frac{\left(b-a\right)}{2}(x+1)$.

**The** **advantages of the method**

- Representing Lagrange polynomials in matrix form improves computational efficiency and simplifies coding.
- The Fejér quadrature with dilation avoids specialized oscillatory integral techniques while maintaining accuracy.
- The method maintains high accuracy using standard numerical integration.
- The method enhances computational speed and ease of implementation.

**The** **disadvantages of the method**

- Increasing the parameter *ω* requires more quadrature points, reducing efficiency.
- Extremely oscillatory solutions decrease the accuracy due to limited quadrature resolution.

In summary, the matrix Lagrange basis and Fejér quadrature offer advantages in accuracy, speed, and coding simplicity. The main limitations occur for very large *ω* values, where efficiency degrades, and for solutions with sharp oscillations, where accuracy declines.

This paper is organized as follows: In Section 2, we introduce the basic concepts and formulas of Chebyshev polynomials and the Fej’er quadrature formula. In Section 3, we derive a method to solve integral Eq (2). In Section 4, we derive convergence rates for the proposed method. In Section 5, we present some numerical examples to demonstrate the efficiency and accuracy of the proposed method.

## 2 Chebyshev polynomials and Fejér quadrature formula

In this section, the basic properties of Chebyshev polynomials and the Fejér quadrature formula are introduced, for details see [24, 25].

### 2.1 Chebyshev polynomials and the Lagrange interpolation process

The Chebyshev polynomial *T*_*n*_(*x*) of the first kind of degree *n* is defined by the relation

$$\begin{array}{c} T_{n}\left(x\right)=\text{cos}\left(n\theta \right),\;when\;x=\text{cos}\left(\theta \right). \end{array}$$

Using the trigonometric identity

$$\begin{array}{c} \text{cos}\;n\theta +\text{cos}(n-2)\theta =2\;\text{cos}\;\theta \;\text{cos}(n-1)\theta , \end{array}$$

the fundamental recurrence relation is obtained

$$\begin{array}{c} T_{n}\left(x\right)=2xT_{n-1}\left(x\right)-T_{n-2}\left(x\right),\;n=2,3,\ldots . \end{array}$$

The initial conditions

$$\begin{array}{c} T_{0}\left(x\right)=1,\;T_{1}\left(x\right)=x, \end{array}$$

efficiently generate all *T*_*n*_(*x*) recursively.

The Chebyshev polynomial *T*_*n*_(*x*) has *n* roots located at

$$\begin{array}{c} x_{k}=\text{cos}\;\theta_{k}=\text{cos}\;(\frac{(2k-1)\pi }{2n}),\;k=1,\ldots ,n \end{array}$$

which are commonly referred to as the Chebyshev nodes.

Denote ${\left\{x_{k}\right\}}_{k=1}^{n}$ as the zeros of *T*_*n*_(*x*), the approximating Lagrange polynomial based on these nodes can be expressed as

$$\begin{array}{c} L_{n}\left(f,x\right)=\sum_{k=1}^{n}\ell_{k}\left(x\right)f(x_{k}), \end{array}$$

where *ℓ*_*k*_ is the *k*-th fundamental Lagrange polynomial defined as

$$\begin{array}{c} \ell_{k}\left(x\right)=\prod_{\begin{array}{c} i=1\\ i\neq k \end{array}}^{n}\frac{\left(x-x_{i}\right)}{\left(x_{k}-x_{i}\right)}=\frac{T_{n}\left(x\right)}{T_{n}^{\prime }(x_{k})(x-x_{k})}. \end{array}$$

On the other hand, the approximating polynomial $L_{n}\left(f,x\right)$ to the function *f*(*x*) can be expressed as

$$\begin{array}{c} L_{n}\left(f,x\right)=\sum_{k=0}^{n-1}a_{k}T_{k}\left(x\right), \end{array}$$

where

$$\begin{array}{c} a_{k}=\{\begin{array}{cc} \frac{2}{n}\sum_{m=1}^{n}f(x_{m})T_{k}(x_{m}) & \;\text{if}\;k>0\\ \frac{1}{n}\sum_{m=1}^{n}f(x_{m}) & \;\text{if}\;k=0, \end{array} \end{array}$$

and *x*_*m*_ are the roots of *T*_*n*_(*x*). By changing the order of summation, $L_{n}\left(f,x\right)$ can be written in the form of

$$\begin{array}{c} L_{n}\left(f,x\right)=\sum_{m=1}^{n}f(x_{m})S_{n}(x_{m},x), \end{array}$$

where

$$\begin{array}{ccc} S_{n}\left(x,y\right) & = & \frac{1}{n}+\frac{2}{n}\sum_{k=1}^{n-1}T_{k}\left(x\right)T_{k}\left(y\right)\\ & = & \frac{2}{n}\left[T_{0}\left(x\right),T_{1}\left(x\right),\cdots ,T_{n-1}\left(x\right)\right]\cdot {\left[T_{0}\left(y\right),T_{1}\left(y\right),\cdots ,T_{n-1}\left(y\right)\right]}^{T}-\frac{1}{n}. \end{array}$$

Using the matrix form can improve computational efficiency.

### 2.2 Fejér quadrature formula

This subsection introduces basic concepts and formulas for interpolatory integration formulas using Chebyshev nodes. For more details, see [25]. For a continuous function *f* on [−1, 1], we can approximate the integral by sums.

Let

$$\begin{array}{c} I=I\left(f\right)=\int_{-1}^{1}f\left(x\right)dx. \end{array}$$

This integral can be approximated as

$$\begin{array}{c} I_{n}=I_{n}\left(f\right)=\sum_{k=1}^{n}w_{k}f(x_{k}). \end{array}$$

For interpolatory rules using Chebyshev nodes, *w*_*k*_ is given by

$$\begin{array}{c} w_{k}=\int_{-1}^{1}\frac{T_{n}\left(x\right)}{T_{n}^{\prime }(x_{k})(x-x_{k})}dx. \end{array}$$ (3)

Using the Christoffel-Darboux formula for *T*_*n*_(*x*) gives

$$\begin{array}{c} 1+2\sum_{m=1}^{n-1}T_{m}\left(x\right)T_{m}(x_{k})=-\frac{T_{n}\left(x\right)T_{n+1}(x_{k})}{x-x_{k}}. \end{array}$$

Eq (3) can be written as

$$\begin{array}{c} w_{k}=-\frac{2}{T_{n}^{\prime }(x_{k})T_{n+1}(x_{k})}\{1+\sum_{m=1}^{n-1}T_{m}(x_{k})\int_{-1}^{1}T_{m}\left(x\right)dx\}. \end{array}$$ (4)

From $T_{n}^{\prime }\left(\text{cos}\;\theta \right)=n\left(\text{sin}\;n\theta \right)/\text{sin}\;\theta$, we have

$$\begin{array}{c} \begin{array}{c} T_{n}^{\prime }(x_{k})=T_{n}^{\prime }(\text{cos}\;\theta_{k})={\left(-1\right)}^{k-1}n/\text{sin}\;\theta_{k},\\ T_{n+1}(x_{k})=\text{cos}\;\left(n+1\right)\theta_{k}={\left(-1\right)}^{k}\;\text{sin}\;\theta_{k}.\\ \int_{-1}^{1}T_{m}\left(x\right)dx=\int_{0}^{\pi }\;\text{cos}\;m\theta \;\text{sin}\;\theta d\theta =\{\begin{array}{cc} 2/(1-m^{2}), & m\;\text{even,}\;\\ 0, & m\;\text{odd.}\; \end{array} \end{array} \end{array}$$

Substituting these formulas into (4) leads to

$$\begin{array}{c} w_{k}=\frac{2}{n}\{1-2\sum_{m=1}^{\left[n/2\right]}\frac{\text{cos}\;(2m\theta_{k})}{4m^{2}-1}\}. \end{array}$$ (5)

Therefore, Fejér’s integration formula is

$$\begin{array}{c} \int_{-1}^{1}f\left(x\right)dx=\sum_{k=1}^{n}w_{k}f(\text{cos}\frac{2k-1}{2n}\pi ), \end{array}$$ (6)

where *w*_*k*_ defined by (5).

## 3 Nyström-type method for Volterra integral equations

Define operator $V:C^{0}\left(\left[-1,1\right]\right)\to C^{0}\left(\left[-1,1\right]\right)$ as

$$\begin{array}{c} \left(Vy\right)\left(x\right)=\int_{-1}^{x}K\left(\omega \left(x-t\right)\right)y\left(t\right)dt, \end{array}$$

then the Eq (2) can be written in the operator form

$$\begin{array}{c} (I+V)y=f. \end{array}$$ (7)

To represent the unknown function *y*(*x*), we define the Lagrange polynomial based on Chebyshev nodes as follows

$$\begin{array}{c} L_{n}\left(y,x\right)=\sum_{k=1}^{n}S_{n}(x_{k},x)y(x_{k}) \end{array}$$

By substituting this expression into (7), we obtain the following equation

$$\begin{array}{c} \sum_{k=1}^{n}S_{n}(x_{k},x)y(x_{k})+\sum_{k=1}^{n}y\left(x_{k}\right)\int_{-1}^{x}K\left(\omega \left(x-t\right)\right)S_{n}(x_{k},t)dt=f\left(x\right). \end{array}$$

When collocating at the zeros *x*_*k*_ of the Chebyshev polynomials, we derive the linear system of equations

$$\begin{array}{c} \sum_{j=1}^{n}[\delta_{ij}+c_{j}(x_{k})]y_{j}=f(x_{k}),\;k=1,\ldots ,n. \end{array}$$

Here, *y*_*j*_ = *y*(*x*_*j*_) represents the unknowns, and the coefficients *c*_*j*_(*x*_*k*_) are defined as follows

$$\begin{array}{c} c_{j}\left(x\right)=\int_{-1}^{x}S_{n}(x_{j},t)K\left(\omega \left(x-t\right)\right)dt,\;j=1,2,\ldots ,n. \end{array}$$ (8)

The preceding system system of order *n* × *n* can be expressed in matrix form as follows

$$\begin{array}{c} (I_{n}+A_{n})Y=f, \end{array}$$ (9)

where $[A_{n}]={\left[c_{j}(x_{k})\right]}_{j,k=1}^{n}$, $Y={\left[y_{j}\right]}_{j=1}^{n}$, and $f={\left[f(x_{k})\right]}_{k=1}^{n}$.

Once the linear system of Eq (9) has been solved, we can calculate the Nyström interpolant as follows

$$\begin{array}{c} \hat{y}\left(x\right)=f\left(x\right)-\sum_{j=1}^{n}c_{j}\left(x\right)y_{j}. \end{array}$$ (10)

In general, the coefficients *c*_*j*_(*x*_*k*_) cannot be computed analytically. The next question is how to efficiently calculate *c*_*j*_(*x*_*k*_). In [23], a method known as the “dilated product quadrature formula” is introduced. This method involves expanding the interval and subdividing it into numerous smaller intervals to ensure that the integrand function on each small interval is non-oscillatory. We apply the same technique and consider Fejér quadrature formula.

With the change of variable *t* = *z*/*ω*, we can express

$$\begin{array}{c} c_{j}\left(x\right)=\frac{1}{\omega }\int_{-\omega }^{\omega x}S_{n}(x_{j},\frac{z}{\omega })K\left(\omega x-z\right)dz. \end{array}$$

Dividing the interval into N subintervals each of length *δ* gives

$$\begin{array}{c} c_{j}\left(x\right)=\frac{1}{\omega }\sum_{\iota =1}^{N}\int_{-\omega +(\iota -1)\delta }^{-\omega +\iota \delta }S_{n}(x_{j},\frac{z}{\omega })K\left(\omega x-z\right)dz. \end{array}$$

By remapping each integral to [−1, 1] with the transformation

$$\begin{array}{c} t=\varphi_{\iota }\left(z\right)=\frac{2}{\delta }\left(z+\omega \right)+1-2\iota , \end{array}$$

each integral can be approximated using the n-point Fejér quadrature formula.

$$\begin{array}{c} c_{j}^{n}\left(x\right)=\frac{\delta }{2\omega }\sum_{\iota =1}^{N}\sum_{k=1}^{n}w_{k}S_{n}(x_{j},\frac{\varphi_{\iota }^{-1}\left(t_{k}\right)}{\omega })K\left(\omega x-\varphi_{\iota }^{-1}\left(t_{k}\right)\right). \end{array}$$ (11)

## 4 Convergence and error analysis

By utilizing the asymptotic behavior of the coefficients in the Chebyshev series expansion of function and by considering the aliasing errors of Fejér’s first and second rules for approximating integrals, Xiang derive improved convergence rates for Fejér’s rules. The key innovations involve utilizing asymptotics to derive tighter bounds on aliasing errors and combining this information with existing error analysis to achieve overall sharper rates, as stated in the following lemma.

**Lemma 1** [26] *Suppose* *f*(*x*) *has an absolutely continuous* (*k* − 1)*st derivative f*^*k*−1^ *on* [−1, 1] *and a kth derivative f*^*k*^ *of bounded variation V*_*k*_ *for some* *k* ≥ 1. *Then for n* ≥ max{2, *k* + 1}

$$\begin{array}{c} |I\left[f\right]-I_{n}\left[f\right]|\leq \frac{2V_{k}\left(1+2^{-k}\right)\zeta \left(k+1\right)}{\pi n(n-1)\cdots (n-k)} \end{array}$$ (12)

*where* $I\left[f\right]=\int_{-1}^{1}f\left(x\right)dx$, *I*_*n*_[*f*] *denotes the (n + 1)-point Fejér’s first rules and* $\zeta \left(k+1\right)=\sum_{p=1}^{\infty }\frac{1}{p^{k+1}}$ *denotes the Riemann zeta function*.

Let *c*_*j*_(*x*) and $c_{j}^{n}\left(x\right)$ be the coefficients defined in (8) and (11), respectively. Assume that $S_{n}(x,\frac{\varphi_{\iota }^{-1}\left(t\right)}{\omega })K\left(\omega x-\varphi_{\iota }^{-1}\left(t\right)\right)$ has an absolutely continuous (*k* − 1)st derivative on [−1, 1] and a *k*th derivative

$$\frac{\partial^{k}(S_{n}(x,\frac{\varphi_{\iota }^{-1}\left(t\right)}{\omega })K\left(\omega x-\varphi_{\iota }^{-1}\left(t\right)\right))}{\partial t^{k}}$$

of bounded variation *V*_*k*_ for some *k* ≥ 1. Then,

$$\begin{array}{c} |c_{j}\left(x\right)-c_{j}^{n}\left(x\right)|\leq \frac{2V_{k}\left(1+2^{-k}\right)\zeta \left(k+1\right)}{\pi n(n-1)\cdots (n-k)}. \end{array}$$ (13)

Define

$$\begin{array}{ccc} \left(V_{m}y\right)\left(x\right) & = & \sum_{j=1}^{m}c_{j}\left(x\right)y\left(t_{j}\right)\\ \left(V_{m}^{n}y\right)\left(x\right) & = & \sum_{j=1}^{m}c_{j}^{n}\left(x\right)y\left(t_{j}\right)\\ \phi (t) & = & \sqrt{\left(1-t)(t+1\right)}. \end{array}$$

The corresponding approximate equation can then be written as

$$\begin{array}{c} \left(I+V_{n}^{m}\right)y_{m}^{n}=f. \end{array}$$ (14)

Denote

$$\begin{array}{c} |e_{m}^{n}\left(y,x\right)|=|\left(Vy\right)\left(x\right)-\left(V_{m}^{n}y\right)\left(x\right)\left|\leq \right|\left(Vy\right)\left(x\right)-\left(V_{m}y\right)\left(x\right)\left|+\right|\left(V_{m}y\right)\left(x\right)-\left(V_{m}^{n}y\right)\left(x\right)|. \end{array}$$

Assuming that the kernel function *K* satisfies the following conditions

$$\begin{array}{c} \underset{x\in [-1,1]}{\text{sup}}\int_{-1}^{1}|K\left(x,t\right)|\text{log}(2+|K\left(x,t\right)|)dt<\infty ,\;\;\underset{x\in [-1,1]}{\text{sup}}\;\frac{\left|K(x,t)\right|}{\sqrt{\phi (t)}}dt<\infty . \end{array}$$ (15)

Also,

$$\begin{array}{c} \underset{x,t\in [-1,1]}{\text{max}}\;|\frac{\partial^{r}K\left(x,t\right)}{\partial t^{r}}\phi^{r}\left(t\right)|<+\infty . \end{array}$$ (16)

By applying Theorem 5.1.12 in [27] we can obtain

$$\begin{array}{ccc} |e_{m}^{n}\left(y,x\right)| & \leq & CE_{m-1}\left(y\right)+\sum_{j=1}^{m}|c_{j}\left(x\right)-c_{j}^{n}\left(x\right)||y\left(t_{j}\right)|\\ & \leq & CE_{m-1}\left(y\right)+{\left\|y\right\|}_{\infty }\sum_{j=1}^{m}\left|c_{j}\left(x\right)-c_{j}^{n}\left(x\right)\right| \end{array}$$ (17)

where $E_{m}\left(y\right)={\text{inf}}_{P_{m}\in P_{m}}{\left\|y-P_{m}\right\|}_{\infty }$ and $P_{m}$ denotes the space of the algebraic polynomials of degree at most *m*.

**Theorem 1** *Denote by* *y* *the unique solution of* Eq (7) *and by* $y_{m}^{n}$ *the unique solution of* (14). *Then, if f*(*x*) ∈ *C*^*r*^([−1, 1]) *and the kernel K satisfies the conditions* (15) *and* (16), *the following error estimate holds true*

$$\begin{array}{c} \|y-y_{m}^{n}{\|}_{\infty }=O\left(\frac{1}{m^{r}}\right), \end{array}$$

*where the constants in* O *do not depend on* *n* *and m*.

**Proof** *From* (7) *and* (14) *we deduce that*

$$\begin{array}{c} \|y-y_{m}^{n}{\|}_{\infty }\leq \|\left(V-V_{m}^{n}\right)y{)\|}_{\infty }, \end{array}$$

*so that by applying* (17) *and* (13) *and the assumptions we obtain the result*.

## 5 Numerical examples

This section provides several numerical examples to demonstrate the effectiveness and accuracy of the proposed algorithm. For each example, *c*_*j*_(*x*) is computed using the 20-point Fejér quadrature formula, and the solution is approximated using the Nyström interpolant in (10). The absolute error

$$\varepsilon_{n}^{20}\left(x\right)=|y\left(x\right)-{\hat{y}}_{n}^{20}\left(x\right)|$$

is used to measure the algorithm’s accuracy, where *y*(*x*) is the exact solution. For Example 2 and 3, we consider the precise approximations generated by the product rule with *m* = 750 and calculate the errors

$$\begin{array}{c} \varepsilon_{750,n}^{20}\left(s\right)=|y_{750}^{20}\left(x\right)-y_{n}^{20}\left(x\right)|. \end{array}$$

Example 1. Consider the following integral equation

$$\begin{array}{c} y\left(x\right)+\int_{-1}^{x}\text{cos}\left(\omega \left(t-x\right)\right)y\left(t\right)dt=f\left(x\right),\;x\in \left[-1,1\right], \end{array}$$

where *y*(*x*) = *e*^*x*^ is the exact solution and the right-hand side *f*(*x*) is given by

$$f\left(x\right)=e^{x}+{\left(e\left(1+\omega^{2}\right)\right)}^{-1}(e^{x+1}-\text{cos}\left(\omega \left(1+x\right)+\omega \;\text{sin}\left(\omega \left(1+x\right)\right)\right).$$

**Fig 1:**
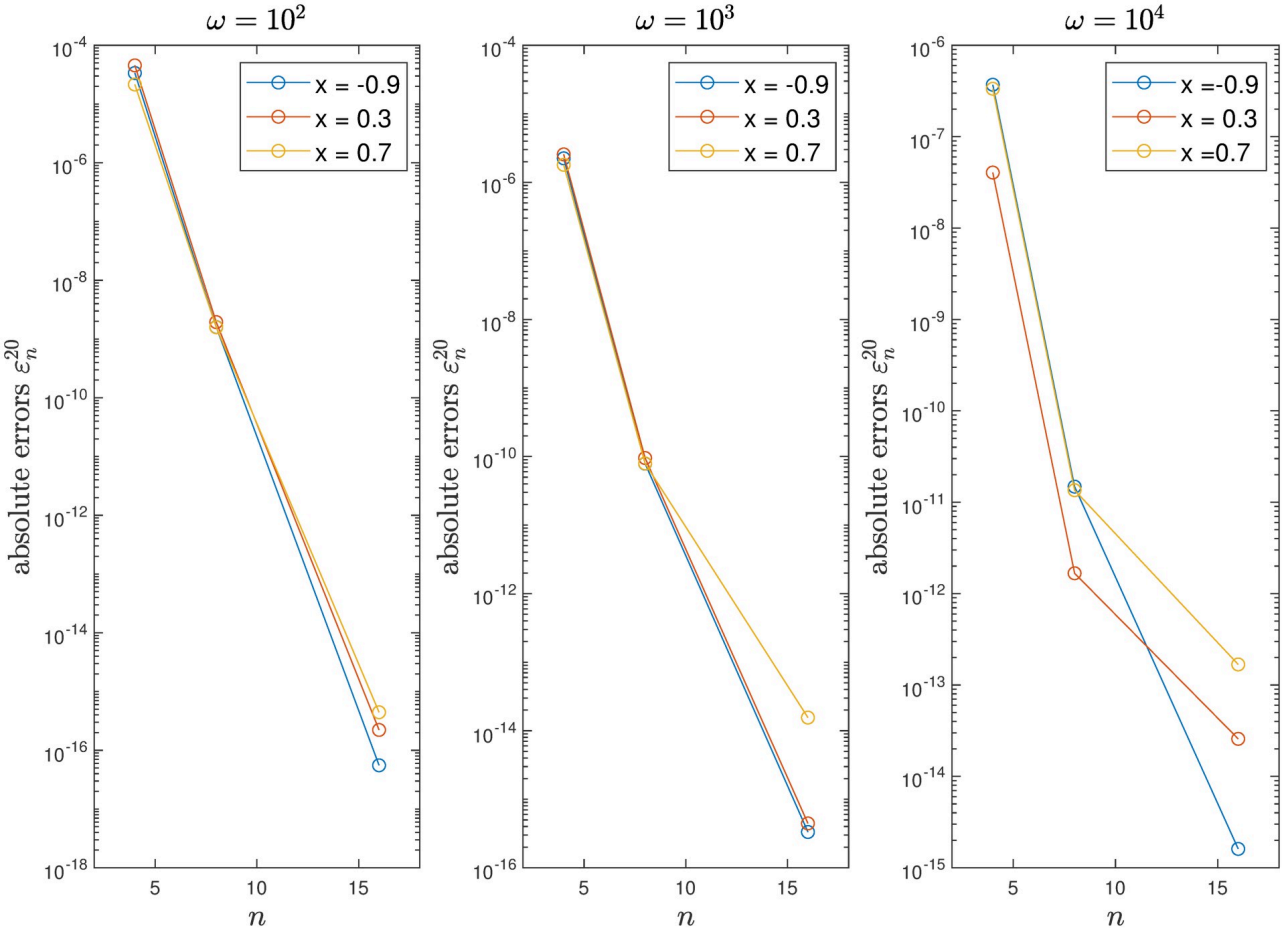
Convergence of the method for Example 1.

Since the kernel function, right-hand side, and solution are all analytic and non-oscillatory for Example 1, convergence is very rapid as seen in Table 1 and Fig 1. For the same *n* and *ω* values, the computing time (e.g. 0.1549s for *n* = 16, *ω* = 10^4^) is approximately 10 times less than the 1.5381s reported in [23], demonstrating the improved efficiency of the proposed method. The calculated condition number of the matrix $I_{n}+A_{n}$ also indicates the system is extremely well-conditioned.

**Table 1:** Numerical result for Example 1.

Methods	*ω*	*n*	CPU times	$\varepsilon_{n}^{2}0\left(-0.9\right)$	$\varepsilon_{n}^{2}0\left(0.3\right)$	$\varepsilon_{n}^{2}0\left(0.7\right)$	$\text{cond}(I_{n}+A_{n})$
Our method	10^2^	4	0.004532	3.3748 × 10^−5^	4.5491 × 10^−5^	2.1486 × 10^−5^	1.02
		8	0.001492	1.5980 × 10^−9^	1.9408 × 10^−9^	1.5857 × 10^−9^	1.03
		16	0.004065	5.5511 × 10^−17^	2.2204 × 10^−16^	4.4409 × 10^−16^	1.04
	10^3^	4	0.016408	2.2432 × 10^−6^	2.5593 × 10^−6^	1.8020 × 10^−6^	1.00
		8	0.016871	7.8768 × 10^−11^	9.5104 × 10^−11^	7.9248 × 10^−11^	1.00
		16	0.018557	3.3307 × 10^−16^	4.4409 × 10^−16^	1.5543 × 10^−14^	1.00
	10^4^	4	0.058717	3.6976 × 10^−7^	4.0498 × 10^−8^	3.3414 × 10^−7^	1.00
		8	0.096697	1.4816 × 10^−11^	1.6709 × 10^−12^	1.3537 × 10^−11^	1.00
		16	0.165109	1.6098 × 10^−15^	2.5757 × 10^−14^	1.6787 × 10^−13^	1.00
Method in [23]	10^2^	4	0.0120	7.33 × 10^−5^	7.87 × 10^−5^	3.58 × 10^−5^	1.05
		8	0.0074	4.95 × 10^−9^	4.80 × 10^−9^	3.03 × 10^−9^	1.09
		16	0.0178	M.P.	6.66 × 10^−16^	4.44 × 10^−16^	1.16
	10^3^	4	0.0336	7.09 × 10^−6^	4.67 × 10^−6^	3.22 × 10^−6^	1.01
		8	0.0563	3.85 × 10^−10^	2.50 × 10^−10^	1.91 × 10^−10^	1.01
		16	0.1530	M.P.	4.44 × 10^−16^	1.78 × 10^−15^	1.01
	10^4^	4	0.2365	7.56 × 10^−7^	7.34 × 10^−8^	6.08 × 10^−7^	1.00
		8	0.5445	4.21 × 10^−11^	4.19 × 10^−12^	3.39 × 10^−11^	1.00
		16	1.5381	6.99 × 10^−15^	1.58 × 10^−14^	3.51 × 10^−14^	1.00

Example 2. Let us consider the following integral equation

$$\begin{array}{c} y\left(x\right)+\int_{-1}^{x}\frac{J_{1}\left(\omega \left(t-x\right)\right)}{\omega (t-x)}y\left(t\right)dt={\left|\text{sin}\left(x+3\right)\right|}^{\frac{11}{2}},\;x\in \left[-1,1\right], \end{array}$$

whose exact solution is not known.

Example 3. Let us consider the following integral equation

$$\begin{array}{c} y\left(x\right)+\int_{-1}^{x}\frac{\text{sin}(\omega (t-x))}{\omega (t-x)}y\left(t\right)dt=\left(3x^{2}+1\right){\left|x\right|}^{\frac{5}{2}},\;x\in \left[-1,1\right], \end{array}$$

whose exact solution is not known.

**Fig 2:**
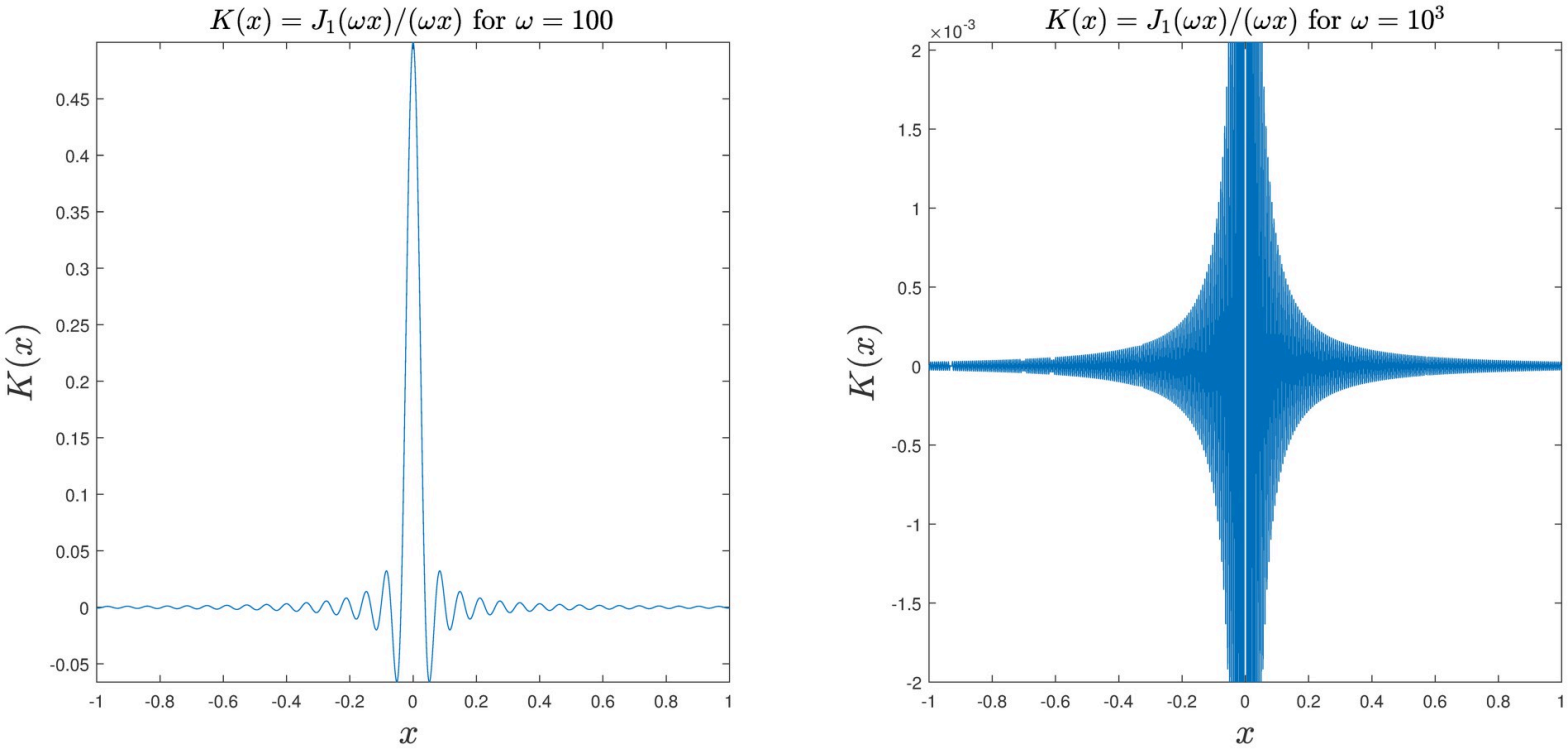
Oscillatory behavior of the integrand.

**Fig 3:**
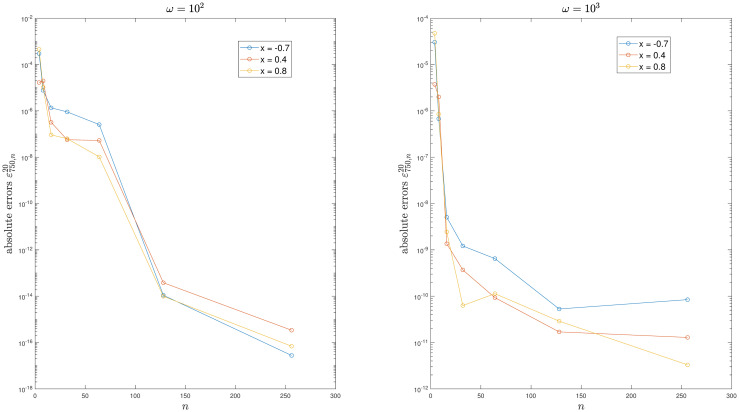
Convergence of the method for Example 2.

**Fig 4:**
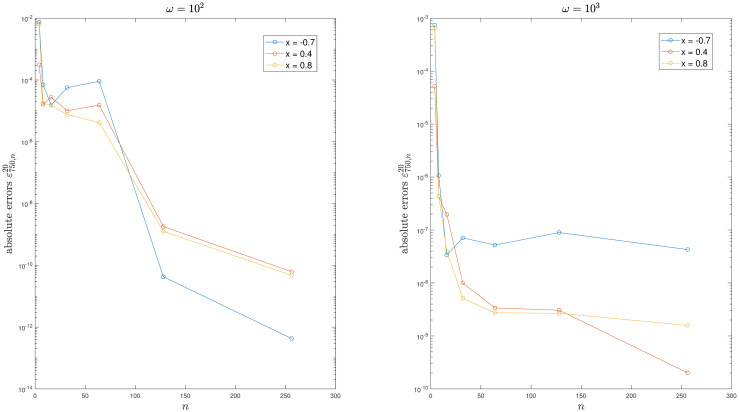
Convergence of the method for Example 3.

For example 2, Fig 2 illustrates that the kernel function exhibits increased oscillations as *ω* increases. Tables 2 to 3 and Figs 3 to 4 demonstrate the convergence of the method. It is easy to see that the scheme is efficient. Moreover, the condition number of the system does not depend on *n*.

**Table 2:** Numerical result for Example 2.

Methods	*ω*	*n*	CPU times	$\varepsilon_{n}^{20}\left(-0.7\right)$	$\varepsilon_{n}^{20}\left(0.4\right)$	$\varepsilon_{n}^{20}\left(0.8\right)$	$\text{cond}(I_{n}+A_{n})$
Our method	10^2^	4	0.009918	2.9537 × 10^−4^	1.6684 × 10^−5^	4.8463 × 10^−4^	1.00
		8	0.006954	7.6694 × 10^−6^	1.9701 × 10^−5^	1.0352 × 10^−5^	1.00
		16	0.005332	1.3510 × 10^−6^	3.2085 × 10^−7^	9.2008 × 10^−8^	1.01
		32	0.011632	9.0356 × 10^−7^	5.6635 × 10^−8^	6.3035 × 10^−8^	1.01
		64	0.047521	2.5531 × 10^−7^	5.3077 × 10^−8^	1.0311 × 10^−8^	1.01
		128	0.189143	1.1019 × 10^−14^	3.8232 × 10^−14^	9.9087 × 10^−15^	1.01
		256	0.663162	2.7756 × 10^−17^	3.4066 × 10^−16^	6.9389 × 10^−17^	1.01
	10^3^	4	0.021445	3.0360 × 10^−5^	3.7172 × 10^−6^	4.7200 × 10^−5^	1.00
		8	0.024735	6.7551 × 10^−7^	1.9997 × 10^−6^	8.4921 × 10^−7^	1.00
		16	0.039830	5.0625 × 10^−9^	1.3580 × 10^−9^	2.4423 × 10^−9^	1.00
		32	0.091650	1.2145 × 10^−9^	3.6956 × 10^−10^	6.2943 × 10^−11^	1.00
		64	0.395427	6.4832 × 10^−10^	9.3056 × 10^−11^	1.1346 × 10^−10^	1.00
		128	1.494718	5.3066 × 10^−11^	1.6988 × 10^−11^	2.8953 × 10^−11^	1.00
		256	6.090813	8.4344 × 10^−11^	1.2893 × 10^−11^	3.3001 × 10^−12^	1.00
Method in [23]	10^2^	4	0.0117	2.59 × 10^−4^	9.13 × 10^−5^	2.72 × 10^−4^	1.00
		8	0.0101	4.50 × 10^−6^	1.56 × 10^−5^	1.75 × 10^−6^	1.02
		16	0.0229	7.79 × 10^−8^	1.11 × 10^−7^	5.80 × 10^−8^	1.04
		32	0.0595	6.43 × 10^−7^	2.60 × 10^−8^	3.26 × 10^−8^	1.05
		64	0.1997	9.14 × 10^−8^	4.13 × 10^−8^	2.20 × 10^−9^	1.05
		128	0.7873	9.58 × 10^−15^	2.80 × 10^−14^	5.59 × 10^−15^	1.04
		256	3.6194	8.33 × 10^−17^	3.35 × 10^−16^	8.33 × 10^−17^	1.08
	10^3^	4	0.0459	2.71 × 10^−5^	1.09 × 10^−5^	2.48 × 10^−5^	1.00
		8	0.0766	5.45 × 10^−7^	1.53 × 10^−6^	6.49 × 10^−8^	1.00
		16	0.1914	5.48 × 10^−9^	6.47 × 10^−10^	3.51 × 10^−9^	1.00
		32	0.5583	1.76 × 10^−10^	6.42 × 10^−11^	3.13 × 10^−11^	1.01
		64	1.9459	1.85 × 10^−10^	1.15 × 10^−11^	3.90 × 10^−11^	1.01
		128	7.8873	1.03 × 10^−11^	1.27 × 10^−12^	2.86 × 10^−11^	1.01
		256	34.8418	1.52 × 10^−11^	1.97 × 10^−11^	1.49 × 10^−12^	1.01

**Table 3:** Numerical result for Example 3.

*ω*	*n*	CPU times	$\varepsilon_{n}^{20}\left(-0.7\right)$	$\varepsilon_{n}^{20}\left(0.4\right)$	$\varepsilon_{n}^{20}\left(0.8\right)$	$\text{cond}(I_{n}+A_{n})$
10^2^	4	0.008531	7.4627 × 10^−3^	3.1078 × 10^−4^	6.4530 × 10^−3^	1.00
	8	0.001441	6.9745 × 10^−5^	1.6461 × 10^−5^	1.7756 × 10^−5^	1.00
	16	0.003166	1.5311 × 10^−5^	2.7722 × 10^−5^	1.5081 × 10^−5^	1.01
	32	0.008148	5.6482 × 10^−5^	1.0068 × 10^−5^	7.5956 × 10^−6^	1.02
	64	0.022078	9.0696 × 10^−5^	1.5366 × 10^−5^	4.1761 × 10^−6^	1.02
	128	0.149627	4.2583 × 10^−11^	1.803 × 10^−9^	1.2585 × 10^−9^	1.02
	256	0.671265	4.2877 × 10^−13^	6.2422 × 10^−11^	4.75531 × 10^−11^	1.02
10^3^	4	0.015664	7.4328 × 10^−4^	5.1706 × 10^−5^	6.3950 × 10^−4^	1.00
	8	0.012255	1.0710 × 10^−6^	4.3219 × 10^−7^	4.3325 × 10^−7^	1.00
	16	0.019191	3.3779 × 10^−8^	1.9741 × 10^−7^	4.0104 × 10^−8^	1.00
	32	0.041037	7.1099 × 10^−8^	1.0028 × 10^−8^	5.1451 × 10^−9^	1.00
	64	0.153366	5.2042 × 10^−8^	3.3800 × 10^−9^	2.7332 × 10^−9^	1.00
	128	1.168353	8.9542 × 10^−8^	3.0644 × 10^−9^	2.6791 × 10^−9^	1.00
	256	6.287524	4.2806 × 10^−8^	2.0122 × 10^−10^	1.5783 × 10^−9^	1.00

## 5 Conclusion

This paper proposes a numerical method for solving highly oscillatory Volterra integral equations. The method combines Chebyshev polynomials, the Nyström approach, and n-point Fejér quadrature with dilation, avoiding reliance on specialized techniques for oscillatory integrals. A key efficiency enhancement is the representation of fundamental Lagrange polynomials in matrix form, improving computational speed and simplifying coding implementation. Numerical results demonstrate that the method retains high accuracy using standard numerical integration, despite the oscillatory nature of the problems. Experiments verify the effectiveness of the proposed technique on test examples. Compared to recent literature, the method provides improved computational efficiency. In summary, this work presents an accurate and computationally advantageous scheme for oscillatory Volterra integral equations. By leveraging these numerical techniques, the proposed Nyström-type method is efficient, straightforward to code, and suitable for practical applications.

## References

[1] Iserles A. and Nørsett S. P. Efficient quadrature of highly oscillatory integrals using derivatives. *Proc. Royal Soc. A*., 461:1383–1399, 2005. doi: 10.1098/rspa.2004.1401

[2] Levin D. Fast integration of rapidly oscillatory integrals. *J. Comp. Appl. Math*., 67:95–101, 1996. doi: 10.1016/0377-0427(94)00118-9

[3] Huybrechs D. and Vandewalle S. A sparse discretization for integral equation formulations of high frequency scattering problems. *SIAM J. Sci.Comput*., 29(6):2305–2328, 2007. doi: 10.1137/060651525

[4] Xiang S. Efficient filon-type methods for $\int_{a}^{b}f\left(x\right)e^{i\omega g(x)}dx$. *Numerische Mathematik*, 105(4):633–658, 2007. doi: 10.1007/s00211-006-0051-0

[5] Xiang S., He G., and Cho Y. On error bounds of filon-clenshaw-curtis quadrature for highly oscillatory integrals. *Advances in Computational Mathematics*, 41:573–597, 2014. doi: 10.1007/s10444-014-9377-9

[6] Xiang S., Chen X., and Wang H. Error bounds for approximation in chebyshev points. *Numer. Math*., 116:463–491, 2010. doi: 10.1007/s00211-010-0309-4

[7] Dominguez V., Graham I., and Smyshlyaev V. Stability and error estimates for filon-clenshaw-curtis rules for highly oscillatory integrals. *IMA J. Numer. Anal*., 31:1253–1280, 2011. doi: 10.1093/imanum/drq036

[8] Dominguez V., Graham I., and Kim T. Filon–clenshaw–curtis rules for highly oscillatory integrals with algebraic singularities and stationary points. *SIAM J. Numer. Anal*., 51:1542–1566, 2013. doi: 10.1137/120884146

[9] Davies Penny J and Duncan Dugald B. Stability and convergence of collocation schemes for retarded potential integral equations. *SIAM journal on numerical analysis*, 42(3):1167–1188, 2004. doi: 10.1137/S0036142901395321

[10] Wang H. Y. and Xiang S. H. Asymptotic expansion and filon-type methods for a volterra integral equation with a highly oscillatory kernel. *IMA Journal of Numerical Analysis*, 31(2):469–490, 2011. doi: 10.1093/imanum/drp048

[11] Xiang S. H. and Brunner H. Efficient methods for volterra integral equations with highly oscillatory bessel kernels. *BIT Numerical Mathematics*, 53(1):241–263, 2013. doi: 10.1007/s10543-012-0399-8

[12] Xiang S. H. and Wu Q. H. Numerical solutions to volterra integral equations of the second kind with oscillatory trigonometric kernels. *Applied Mathematics and Computation*, 223:34–44, 2013. doi: 10.1016/j.amc.2013.07.075

[13] Xiang S. H. and He K. X. On the implementation of discontinuous galerkin methods for volterra integral equations with highly oscillatory bessel kernels. *Applied Mathematics and Computation*, 219(9):4884–4891, 2013. doi: 10.1016/j.amc.2012.10.073

[14] Wu Q. H. On graded meshes for weakly singular volterra integral equations with oscillatory trigonometric kernels. *Journal of Computational and Applied Mathematics*, 263:370–376, 2014. doi: 10.1016/j.cam.2013.12.039

[15] Brunner H. On volterra integral operators with highly oscillatory kernels. *Discrete and Continuous Dynamical Systems*, 34(3):915–929, 2014. doi: 10.3934/dcds.2014.34.915

[16] Ma J. J., Fang C. H., and Xiang S. H. Modified asymptotic orders of the direct filon method for a class of volterra integral equations. *Journal of Computational and Applied Mathematics*, 281:120–125, 2015. doi: 10.1016/j.cam.2014.12.010

[17] Brunner H., Ma Y. Y., and Xu Y. S. The oscillation of solutions of volterra integral and integro-differential equations with highly oscillatory kernels. *Journal of Integral Equations and Applications*, 27(4):455–487, 2015. doi: 10.1216/JIE-2015-27-4-455

[18] Xiang S. H., Li B., and Liu G. D. On efficient computation of highly oscillatory retarded potential integral equations. *International Journal of Computer Mathematics*, 95(11):2240–2255, 2018. doi: 10.1080/00207160.2017.1380192

[19] Zhang Q. Y. and Xiang S. H. On fast multipole methods for volterra integral equations with highly oscillatory kernels. *Journal of Computational and Applied Mathematics*, 348:535–554, 2019. doi: 10.1016/j.cam.2018.09.009

[20] Wu Qinghua and Sun Mengjun. Numerical steepest descent method for hankel type of hypersingular oscillatory integrals in electromagnetic scattering problems. *Advances in Mathematical Physics*, 2021:8021050, 2021.

[21] Zhao Longbin, Fan Qiongqi, and Ming Wanyuan. Efficient collocation methods for volterra integral equations with highly oscillatory kernel. *Journal of Computational and Applied Mathematics*, 404:113871, 2022. doi: 10.1016/j.cam.2021.113871

[22] Zhao Longbin, Fan Qiongqi, and Wang Sheng. High asymptotic order methods for highly oscillatory integral equations with trigonometric kernels. *Journal of Computational and Applied Mathematics*, 416:114549, 2022. doi: 10.1016/j.cam.2022.114549

[23] Fermo Luisa and Van Der Mee Cornelis. Volterra integral equations with highly oscillatory kernels: a new numerical method with applications. *Electronic Transactions on Numerical Analysis*, 54:333–354, 2021. doi: 10.1553/etna_vol54s333

[24] Mason John C and Handscomb David C. *Chebyshev polynomials*. Chapman and Hall/CRC, 2002.

[25] Davis P. J. and Rabinowitz P. *Methods of numerical integration* 2nd ed. Dover Publications, 2007.

[26] Xiang Shuhuang. On convergence rates of fejér and gauss-chebyshev quadrature rules. *Journal of Mathematical Analysis and Applications*, 405(2):687–699, 2013. doi: 10.1016/j.jmaa.2013.04.027

[27] Mastroianni Giuseppe and Milovanović Gradimir V. *Interpolation processes: Basic theory and applications*. Springer, 2008.

